# An arthroscopic repair technique for meniscal tear using a needle and suture: outside-in transfer all-inside repair

**DOI:** 10.1186/s12891-019-2984-3

**Published:** 2019-12-23

**Authors:** Zhiqiang Wang, Yan Xiong, Xin Tang, Qi Li, Zhong Zhang, Jian Li, Gang Chen

**Affiliations:** 1 Department of Orthopaedic Surgery, West China Hospital, Sichuan University, No. 37, Guoxue Alley, Chengdu, 610041 China; 2 Department of Orthopaedic Surgery, Suining Central Hospital, No. 127, West Desheng Rd., Chuanshan District, Suining, Sichuan, 629000 China

**Keywords:** All-inside, Outside-in, Knee, Meniscus, Suture repair

## Abstract

**Background:**

At present, most repair techniques for meniscal tears fix the meniscus directly over the capsule. This changes the normal anatomy and biomechanics and limits the activity of the meniscus during motion. We introduce an arthroscopic repair technique by suturing the true meniscus tissue without the capsule and subcutaneous tissue.

**Methods:**

After confirmation of a tear, a custom-designed meniscal repair needle first penetrates percutaneously, crossing the capsular portion and the torn meniscus, and exits from the femoral surface of one side of the torn meniscus. Then a No. 2 PDS suture is passed through the needle and retrieved through the arthroscopy portal. Next, the needle is withdrawn to the synovial margin of the meniscus and is reinserted, exiting the femoral surface of the other side of the torn meniscus. The suture is pulled out through the same portal with a grasper. Finally, arthroscopic knotting is performed.

**Results:**

We had 149 cases of meniscal tears repaired with this outside-in transfer all-inside technique since July 2016.

**Conclusions:**

It is a simple, minimally invasive, and economical procedure that is appropriate for most parts of the meniscus except the posterior horn of the lateral meniscus, and it can be used to fix torn meniscus tissue firmly while also preserving the inherent activity of the meniscus.

## Background

Meniscal injuries are one of the most common injuries in sport medicine. The mean annual incidence of meniscal lesions per 10,000 inhabitants was 9.0 in males and 4.2 in females [1]. Meniscectomy increases the risk of developing osteoarthritis (OA) of the knee significantly after 20 years [2]. Meniscal repairs are preferable over partial or total meniscectomies as they aim to restore a functional meniscus and possibly prevent early degenerative changes.

Many meniscal repair techniques such as outside-in, inside-out, and all-inside have been described for the treatment of these tears [3–7]. The outside-in technique is an option for anterior and middle-segment meniscal tears, but one disadvantage of this technique is that an additional 1–2 cm skin incision is required, and knots are tied subcutaneously over the capsule [8]. The gold-standard technique for meniscal repair has been the inside-out technique [9]. Some problems that are specific to this approach include the need for an accessory incision, and potential injuries to the medial saphenous, peroneal nerve, or lateral popliteal neurovascular bundle [10]. The all-inside repair technique can be used to repair the posterior horns and middle segments of the meniscus; however, this technique requires a special meniscal repair device, is expensive, and increases the risk of posterior lateral tears to the neurovascular bundle [11]. Furthermore, several material-related complications have been reported for the all-inside repair technique [12–14]. A systematic review and meta-analysis reported that the pooled rate of meniscal repair failure (reoperation or clinical failure) was 23.1% (131 of 566), which was greater than the five-year failure rates for all of the techniques that were investigated [15]. Many complications associated with meniscal repair have been reported the literature [16–19]. Reducing the risk of surgery and complications has always been the goal of surgeons. The development of a meniscus suture method that can increase meniscal healing and reduce complications is therefore desirable [20].

Here, we report an arthroscopic repair technique in the treatment of meniscal tears for most parts of the meniscus except for the posterior horn of the lateral meniscus. This technique could be used to suture the true meniscus tissue without the capsule and subcutaneous tissue, and could preserve the normal biomechanics of the meniscus during motion.

## Methods

**Fig. 1:**
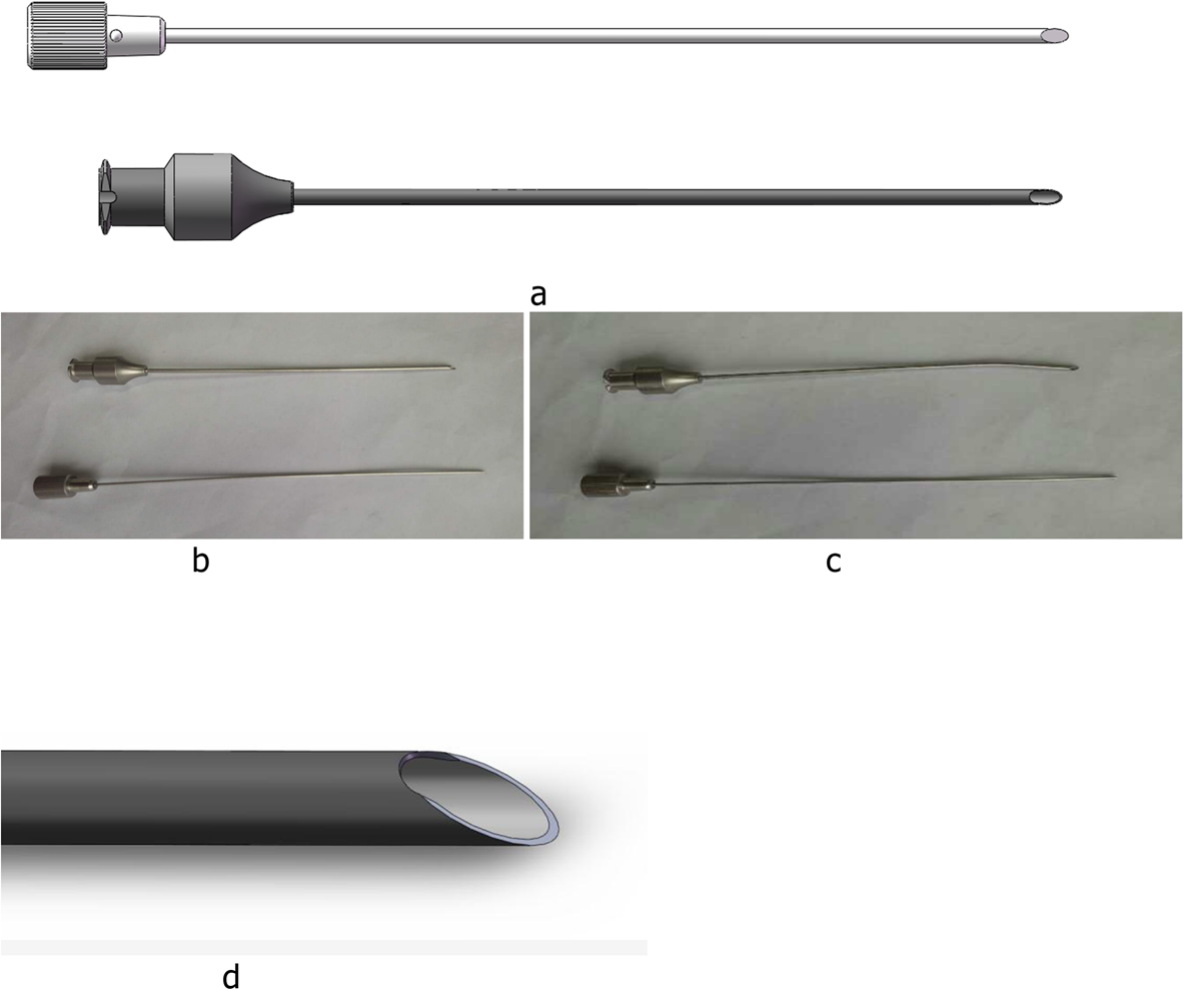
Design drawings (**a**) and physical maps are shown for straight (**b**) and curved (**c**) custom-designed meniscal repair needles. A part of the tip is designed to be blunt, which can protect the suture material from being cut by the beveled needle tip (**d**)

**Fig. 2:**
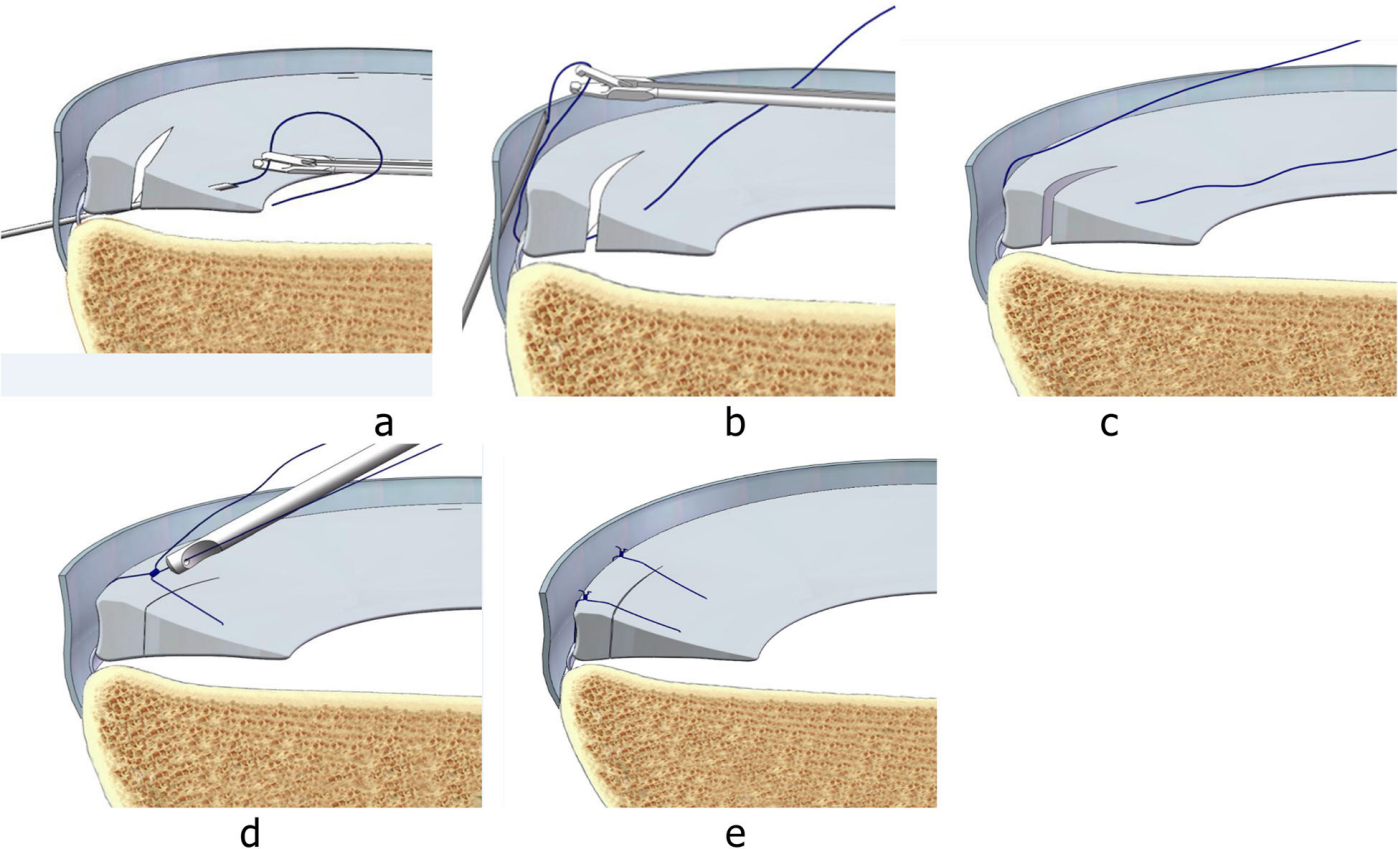
Suturing process (left knee). Meniscus repair penetrating the free edge of the meniscus. Sectional and side views. **a** The needle penetrates the capsular portion, crossing the tibial surface of the meniscus, and then exits the femoral surface of one side of the torn meniscus. **b** The needle is withdrawn to the synovial margin of the meniscus inside the capsule and reinserted upwards, exiting the femoral surface of the other side of the torn meniscus. The suture is pulled out with a grasper. **c** The two limbs of the suture were tensioned. **d** a Samsung Medical Center sliding knot was formed inside the joint with a pusher. **e** Completed stitching

**Fig. 3:**
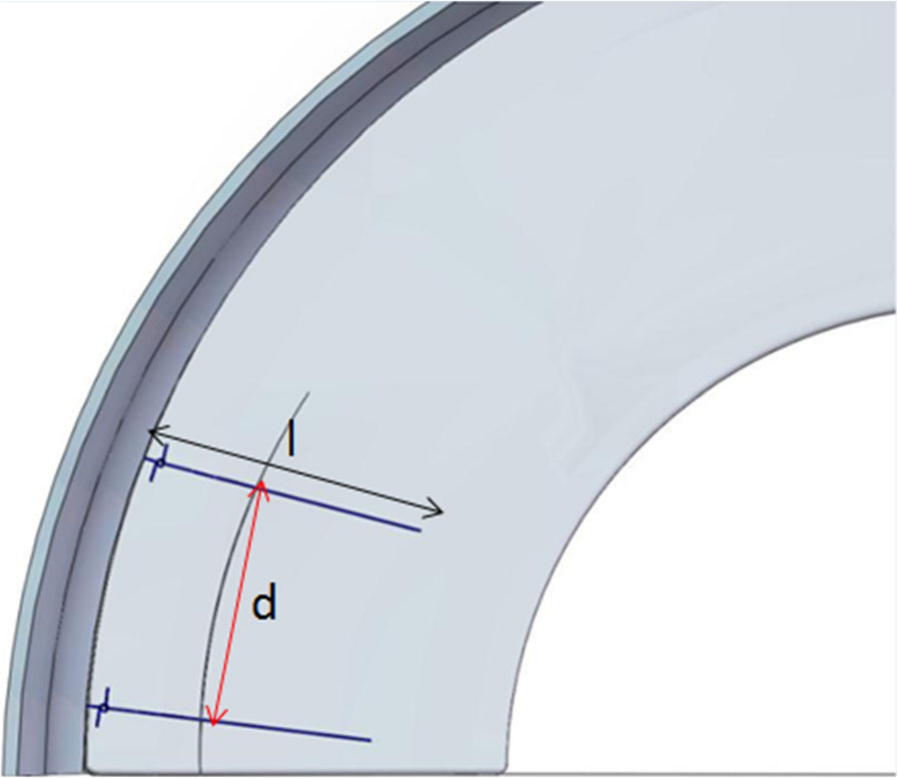
The distance between the two knots (d) is equal to the length of the suture (l).(left knee)


The patient was positioned for standard knee arthroscopy. Routine diagnostic arthroscopy using the standard anterolateral and anteromedial portals was performed. After confirmation of a tear, the meniscal tear site, size, pattern, vascularity, stability, tissue quality, and associated pathology within the knee joint were recorded. Tears located in the red-red and red-white zones can heal, and therefore can be repaired [21]. Before sewing, the tear site was debrided with a 3.5 mm full-radius motorized shaver (Dyonics; Smith & Nephew, Andover, MA, USA) and rasped to encourage healing through the anteromedial portal. Next, the outside-in transfer all-inside repair technique was applied. First, on the skin near the tear site, a custom-designed meniscal repair needle (Fig. 1) penetrated the capsular portion, crossing the tibial surface of the meniscus, and then exited the femoral surface of one side of the torn meniscus. Secondly, a No. 2 PDS (Ethicon, Sommerville, NJ, USA) suture was passed through the meniscal repair needle, and the suture tip was retrieved through the arthroscopy portal using a grasper (Fig. 2a). Thirdly, the needle was withdrawn carefully along the suture to the synovial margin of the meniscus inside the capsule and reinserted upwards without penetrating the meniscus tissue, exiting the femoral surface of the other side of the torn meniscus. The suture that was folded over the needle tip inside the joint was pulled out through the same portal with a grasper (Fig. 2b). Here, the outside-in repair technology was successfully converted to an all inside knotting technology (Fig. 2c). Next, the two limbs of the suture were tensioned simultaneously to achieve satisfactory reduction of the torn meniscus under arthroscopic visualization to form a Samsung Medical Center sliding knot inside the joint (Fig. 2d). Furthermore, additional three or four half-hitch knots with alternating posts on reverse throws were made to secure the knot. The knot was positioned on the synovial side such that the tied knot was positioned towards the peripheral rim portion of the meniscosynovial junction to prevent articular cartilage erosion of the femoral condyle during motion (Fig. 2e). After the knot was tied, the tension was confirmed with a probe via arthroscopic visualization, and a second knot was made. The distance between the two knots is usually 6–8 mm depending on the type of meniscal tear (Fig. 3). The procedure (Additional file 1: Video S1) is repeated as often as necessary to yield a stable meniscal repair. Care was taken to avoid meniscal tissue eversion by appropriately adjusting the knot tension and the suture position. When the procedure is completed, the full range of motion should be performed at least 20 times to assess the reliability of the suture and yield more accurate anatomic reduction. Here, we outlined the vertical suture technique of the meniscus. A horizontal suture technique could be performed in the similar way if necessary.

**Additional file 1: Video S1.** The process of suturing process of the posterior horn of the medial meniscus (left knee) under arthroscope. First, a meniscal repair needle with 2 PDS suture penetrated the capsular portion, crossing the tibial surface of the meniscus, and entered the joint cavity. Secondly, the suture tip was retrieved through the arthroscopy portal using a probe. Thirdly, the needle was withdrawn along the suture to the synovial margin of the meniscus inside the capsule and reinserted upwards without penetrating the meniscus tissue, exiting the femoral surface of the torn meniscus. The suture was pulled out with a probe. Next, the two limbs of the suture were retrieved through the same arthroscopy portal using a grasper and formed a sliding knot inside the joint. Additional knots were made to secure the knot. The suture strands were cut by knot cutter and then he knot was pushed towards the peripheral rim portion of the meniscosynovial junction.


**Fig. 4:**
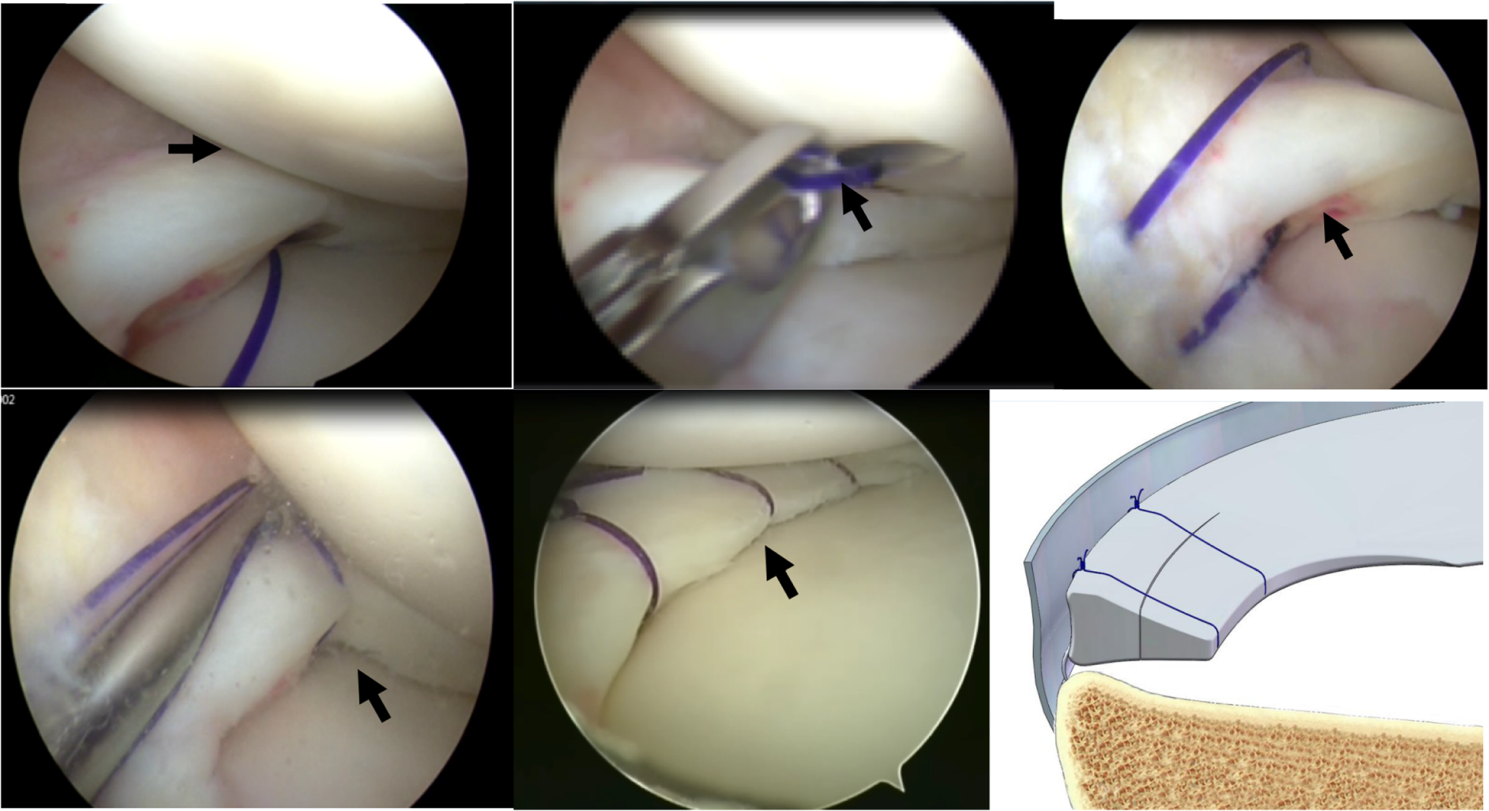
Suturing process of the posterior horn of the medial meniscus (left knee). Meniscus repair without penetrating the meniscus. Arthroscopic view. **a** The needle penetrates the capsular portion, along the tibial surface of the meniscus into the joint cavity. **b** The needle is withdrawn to the synovial margin of the meniscus inside the capsule and reinserted upwards, exiting the femoral surface of the other side of the torn meniscus. The suture is pulled out with a grasper. **c** The two limbs of the suture were tensioned (**d**) a Samsung Medical Center sliding knot was formed inside the joint with a pusher. **e** Completed stitching. **f** Side view

**Fig. 5:**
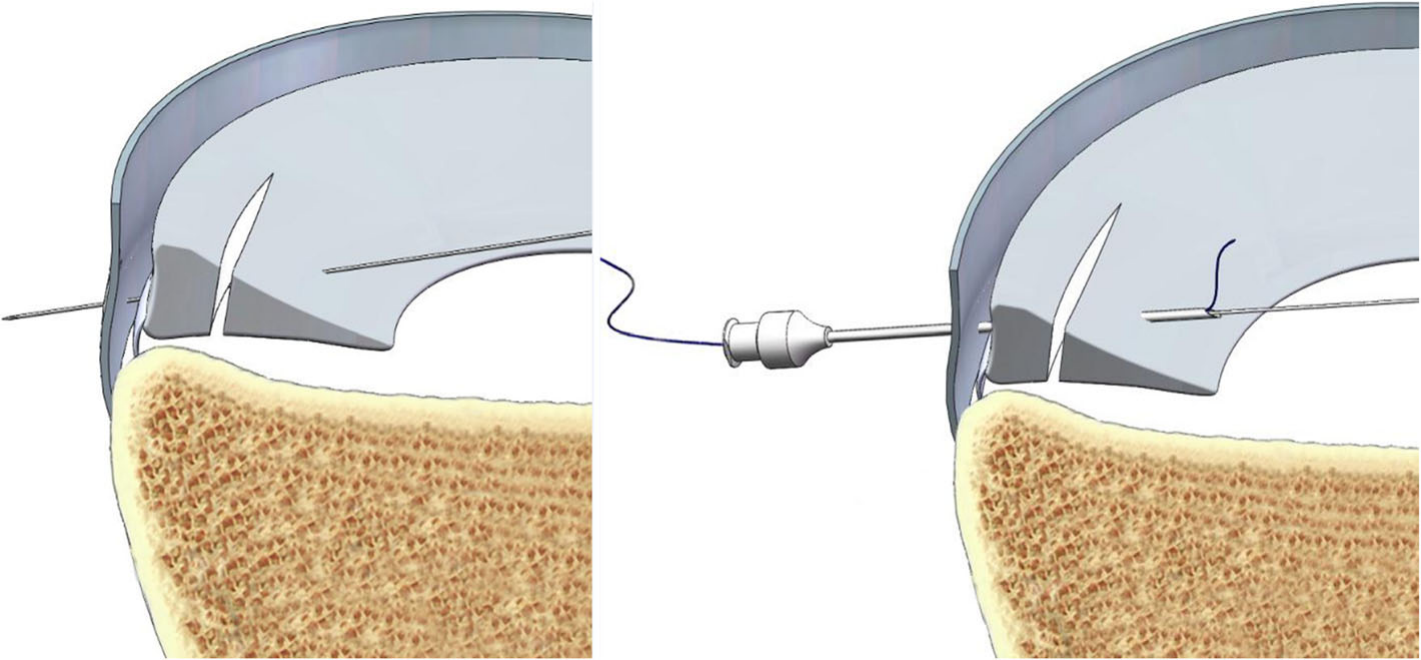
The needle can be guided by a K-wire using the inside-out technique for the repair of tears of the posterior horn of the medial meniscus (left knee)

According to the type of meniscal tear, there are two suture types: one that penetrates the free edge of the meniscus (Fig. 2), and one that does not penetrate the free edge of the meniscus (Fig. 4). For tears of the posterior horn of the medial meniscus, the needle can be guided by a K-wire using the inside-out technique because of the narrow joint space (Fig. 5). This technique should not be used for repairing the posterior horn of the lateral meniscus considering the potential risks of injury to neurovascular structures.

During the postoperative period, all patients were instructed to perform quadriceps-setting exercises. After immobilization for 2 weeks, the knee flexion and extension exercises as well as partial weight-bearing were allowed.

## Results

We had 149 cases of meniscal tears repaired with this outside-in transfer all-inside technique since July 2016. The mean age of the patients was 25.4 years (range, 15–46 years). All patients were able to perform the full range of motion at least 20 times, and the knots did not loosen or slip during surgery. At an average follow-up period of 13 months, there were no cases of post-operative meniscal symptoms and joint line tenderness. A total of 12 patients with anterior or posterior cruciate ligament reconstruction reviewed magnetic resonance image and underwent a second evaluation under arthroscopy, and completely healed meniscal tears were observed.

## Discussion

Meniscal function is essential for normal function of the knee joint. The meniscus is responsible for load transmission and shock absorption of the tibiofemoral joint in the human knee [22]. In addition, it acts as a secondary anteroposterior stabilizer of the knee joint, contributing to proprioception of the knee joint, lubrication, and supply of nutrition to the articular cartilage [22]. The purpose of surgical treatment is to promote meniscal healing and restore the function of the meniscus as much as possible.

Although there are many techniques for meniscus suture technique, the failure rate of the meniscal repair increases from early to long-term follow-up [23], especially for younger patients [24]. The failure rate of medial meniscal sutures has been reported in a systematic review to be 22.3–24.3% for all techniques investigated for 5 years postoperatively [15]. Some anatomical and biomechanical explanations for this high failure rate include blood supply and motion [25–27], but also repair technique factors such as high fixation strength, stability, and tension [28]. The fixation strength required to maintain the meniscus in a reduced position during ambulation and rehabilitation should be considered for long, complex, degenerative tears, and tears, especially in older patients [29]. However, the meniscus has inherent activity during motion of the knee joint [30, 31]. If the meniscus cannot move with knee activity, it may be caught between the condyle of the femur and the plateau of the tibia, thereby causing injury.

The traditional outside-in and inside-out repair techniques do not simply repair the meniscus itself, which also used to suture the meniscus, joint capsule, and subcutaneous tissues together. Excessive extra-articular tissue and nerve endings being knotted will increase the pulling of the meniscus and can even cause pain. Furthermore, these knots could change the normal anatomy and biomechanics, which could limit the activity of the meniscus during motion and increase the chance of meniscal injury. Another disadvantage of this technology is that it will leave a knot outside the capsule. Some patients often complain of knot prominence since the incisions are on the sides of the knee where the extra-capsular tissue layers are thin. The all-inside repair technique with meniscal fixators has been increasingly popular for the posterior horns and middle segments of the meniscus. However, several material-related complications have been reported [12–14]. Parts of the all-inside suture materials had to be removed in some cases because of persistent irritative symptoms [32–35]. Several bioabsorbable devices, which facilitate all-inside meniscal repair (arrows, staples), have been devised, but their use has not been indicated in any tear configuration. We use absorbable PDS suture to sew the meniscus in the present study, which was proved that has superior resistance to failure compared with the other suture types (Ethibond) for vertical mattress technique [36]. In addition, cyst formation has also been reported. Choi et al. [37] found an overall 8% incidence of cyst generation, and Hoffelner et al. [38] found an overall 11.1% incidence in their study. Terai [39] found that cyst formation occurred in 29% of those in which the Fast Fix was used.

The present repair technology is an improvement of the traditional outside-in and all inside repair technology, which could be used to suture or bind the true meniscus tissue, thereby avoiding the joint capsule and subcutaneous tissue being knotted. It could be used to strongly hold the torn meniscus tissue without limiting the motion of the meniscus itself, while also preserving the inherent activity of the meniscus (Table 1).

**Table 1:** the pearls and pitfalls of the present technique

Pearls	Pitfalls
Suturing the true meniscus tissue without excessive extra-articular tissue and nerve endings being knotted could reduce pain after postoperative. Stable fixation during operation Preserving the inherent activity of the meniscus Knotting under arthroscopy avoid meniscus curl and eversion. Arthroscopic visualization assess the proper tension of the suture repair.	The tied knot should be positioned towards the peripheral rim portion of the meniscosynovial junction to prevent articular cartilage erosion of the femoral condyle during motion. A K-wire to guide the meniscal repair needle or pie-crusting tecnique for tears of the posterior horn of the medial meniscus. other techniques such as hook suture should be selected for the posterior horn of the lateral meniscus.

Compared with other methods, this technology has the following advantages (Table 2). First, this is a simple and fast repair technique that only requires a needle and a suture. There is no need to use other expensive materials, which reduces the cost of surgery for patients. Secondly, the current technique is a minimally invasive procedure. The whole process can be completed through the routine arthroscopic portal, without the need to create additional portal and skin incisions. Thirdly, the tension can be adjusted as needed during knotting. Lastly, this is an anatomical suturing procedure, resulting in high fixation strength. This technique could be used to suture the true meniscus tissue without the capsule and subcutaneous tissue, and could preserve inherent activity of the meniscus, decreasing the chance of meniscal re-injury.

**Table 2:** the advantages and disadvantages

Advantages	Disadvantages
Minimally invasive technique Routine arthroscopic approach Low-cost Wide indications Anatomic reduction No knot leave a knot outside the capsule or subcutaneous No material-related complications	High technical requirements Leave a knot inside the joint cavity Potential risks of injury to neurovascular structures for the posterior horn of the lateral meniscus Difficult for posterior horn of the medial meniscus because of narrow space

There were some risks and limitations for this technique. Firstly, there is a potential risk of neurovascular injury if it was used to suture the posterior horn of the lateral meniscus. For this reason, other techniques such as hook suture should be selected for the posterior horn of the lateral meniscus. Secondly, during the process of penetrating, there is a risk that the suture material may be cut by the beveled needle tip. Using our custom-designed meniscal repair needle can effectively avoid this problem. Cartilage injury may be another risk when suturing the posterior horn of the medial meniscus due to narrow joint space. This problem can be effectively avoided by using a K-wire to guide the meniscal repair needle or pie-crusting tecnique. It can ensure ensure accurate penetrate and reduce the risk of cartilage injury during surgery. Of course, there are some other risks such as incision infection, meniscus re-injury, which can be avoided with sterile operations and standard rehabilitation exercises. In addition, according to the type of meniscus tear, this technique can also be used in combination with other methods. For example, we can use this method together with all-inside technique to repair bucket-handle meniscus tear. At last, further prospective clinical studies and biomechanical tests of this technique are needed in the future.

## Conclusions

In conclusion, the technique presented here is suitable for most parts of the meniscus except for the posterior horn of the lateral meniscus. We believe that this technique could provide a good alternative for sports medicine doctors.

## Data Availability

The datasets used and/or analyzed during the current study are available from the corresponding author on reasonable request.
